# Repair of Peripheral Nerve Defects Using a Polyvinylidene Fluoride Channel Containing Nerve Growth Factor and Collagen Gel in Adult Rats

**Published:** 2011-09-23

**Authors:** Hamdollah Delaviz, Abolfazel Faghihi, Alireza Azizzadeh Delshad, Mohamad hadi Bahadori, Jamshid Mohamadi, Amrollah Roozbehi

**Affiliations:** 1. Department of Anatomy, Faculty of Medicine, Yasuj University of Medical Sciences, Cellular and Molecular Research Center, Yasuj, Iran; 2. Department of Anatomy, Faculty of Medicine, Tehran University of Medical Sciences, Tehran, Iran; 3. Department of Anatomy, Faculty of Medicine, Shahed University of Medical Sciences, Tehran, Iran; 4. Department of Anatomy, Faculty of Medicine, Guillan University of Medical Sciences, Guillan, Iran; 5. Departement of Physiology, Faculty of Medicine, Yasuj University of Medical Sciences, Yasuj Iran

**Keywords:** NGF, Sciatic Nerve, Nerve Injury, Spinal Cord, Motor Neuron

## Abstract

**Objective:**

As effectiveness of the autologous graft in the repair of long nerve defects is very limited an effective substitute is needed. This study was conducted to determine the poled polyvinylidene fluoride (PVDF) tube as an alternative to nerve autograft.

**Materials and Methods:**

The left sciatic nerve was transected in 45 male Wistar rats. The animals were then divided randomly into three groups: in an epineural group the nerve was sutured end to end; in an autograft group a 10 mm piece of sciatic nerve was cut, rotated 180° and sutured in the nerve gap; and in a nerve guidance channel group (NGC), PVDF, tube containing nerve growth factor (NGF) and collagen gel was placed in the gap. In a control (n=15) group the sciatic nerve was exposed but not transected. To determine axonal regeneration, retrograde DiI tracer was injected into the gastrocnemius muscle. One week later, retrograde-labeled neurons were counted in the L4-L6 spinal segments and one way ANOVA analysis was performed to compare groups. Neuronal morphology changes were studied by electron microscopy.

**Results:**

Significant statistical decreases in the mean number of labeled motoneurons were observed in all surgical groups compared to the control group; and in the autograft and the NGC groups compared to epinural suture group (p<0.01). No significant difference in the mean number of motoneurons was observed between the autograft and NGC groups. Chromatin condensation, dilated endoplasmic reticulum and large vacuoles were observed in the autograft and NGC groups.

**Conclusion:**

Regarding the positive effects of PVDF tube containing NGF and Collagen gel on the sciatic nerve regeneration, authors suggest that it may be useful in peripheral nerve repair.

## Introduction

 Severe lesions of the peripheral nerves can result in incomplete axonal regeneration and permanent disability in patients(1). Direct nerve repair,such as epineural or fascicular suturing, is applied when there is no gap at the lesion site(2). However, in cases of more severe injury accompanied by long defects in the peripheral nerve a grafting technique is necessary. Autogenous graft is one of the common clinical procedures used to connect the proximal and distal portion of the nerve injury (3-5). However, autogenous grafts are not the most efficient methods for every nerve repair, for example repair of the sciatic nerve. The number of fascicles and length must be correlated with the graft-host interface(2). Therefore, reconstructionof nerve defects remains a surgical challenge(5).So, several biological nerve grafts including arteries, collagen tube, vein and tendon have been tested as conduits for nerve repair (6-9). However, scar infiltration and fibrosis has observed with these organic materials (2). The advantages of synthetic tubes are that they are porous which allows the exchange of nutrients and they have biodegradable properties which lower the inflammatory response (10, 11). Polyvinylidene fluoride (PVDF) is a highly non-reactive and pure thermoplastic fluoropolymer. When polarized, PVDF has piezoelectric properties which have been shown to increase the efficiency of nerve regeneration in vivo and in vitro (12, 13)and PVDFchannels have been shown to support neuronal morphology and decrease cell death when used to repair peripheral nerve injury in rats (14). Furthermore, it can be combined with other therapies to creative a restorative treatment. Collagen gel has mechanical properties that protect neurite elongation in vitro (15)and nerve growth factor(NGF) has been shown to promote nerve regeneration in crushed rat sciatic nerve(16).Collagen gel and NGF reduced cell death in sciatic nerve repair(17)

In the present study our purpose was to evaluate the effect of polarized piezoelectric PVDF channels containing collagen gel and NGF on repair of a 10 mm sciatic nerve defect in rats by tracing and transmission electron-microscope assessment techniques.

## Materials and Methods

### Preparation of polarized piezoelectric polyvinylidene fluoride channel

The polyvinylidene fluoride (Harvard Apparatus Ltd) tube was polarized in the electronics laboratory of Sharif Industrial University as follows: A thin wire inserted into the lumen of the PVDF tube and a circumferential array of steel needles, served as an inner and outer electrode, respectively. The outer needle electrode was connected to the positive output of a voltage supply and the inner electrode was grounded. The voltage output was gradually increased to 21 kv over a 2 hours period and maintained at that level for 12 hours (14).

 The tube was cut into 14 mm pieces, sterilized using 70 % ethanol, filled with 1.28 mg/ml of collagen gel (Roche, Switzerland) and 100 ng/ml of NGF75 (Roche, Switzerland), and then put in a humidified 37℃ incubator for polymerization.

### Animals and surgical procedure

All animal experiments were performed according to the Iranian Council for the Use and Care of Animals Guidelines and were approved by the Animal Research Ethical Committee of Tehran Medical University. Sixty male Wistar r,ats (200-250 g) (Pasteur institute, Tehran, Iran) were divided into four experimental groups; epineural suture, autograft, nerve guidance channel and one control group. Animals were housed in plastic cages with free access to food and water. Their room was maintained at constant temperature of 22-24℃ under 12 hours light/ 12 hours dark cycle. Intraperitoneal ketamine (100 mg/kg) plus xylazine (10 mg/kg) was used as a general anesthetic in all surgical procedures. Under aseptic conditions, the skin and muscles of the back of the left thigh were incised and the sciatic nerve was exposed between the ischial spine and popliteal fossa superior to its bifurcation.

 In the epineural suture group, the left sciatic nerve was transected in the middle of the thigh and then sutured end to end. In the autograft group, 1 cm segment of the nerve was resected and rotated 180° then sutured at the proximal and distal nerve stumps as an autograft. In the nerve guidance channel group (NGC), 1 cm segment of the nerve was resected after which the proximal and distal nerve stumps were inserted into the 14 mm polarized PVDF tube filled with collagen and NGF and fastened with a single 10-0 epineural suture at the proximal and distal ends. In the control animals the sciatic nerve was exposed in the same manner, but the sciatic nerve was not transected.

### Tracing study

Eight weeks after sciatic nerve transection, 10 animals of each group were anesthetized and 40 µl of saturated DiI (1, 1-dioctadecyl-3, 3, 3, 3 -tetramethylindocarbocyanin perchlorat) from Molecular Probes (Leiden, Netherlands; cat. No, D-282) in DMSO was injected at four points in the left gastocnemius muscle. One week later, the animals were deeply anesthetized and perfused transcardialy with 0.9 % heparanized saline followed by fixation with 4 % paraformaldehyde (0.1 M phosphate buffer, pH 7.4). The embedded spinal cord (L4-L6) was dissected out and cryoprotected in 30 % sucrose overnight. Serial 50 µm - thick transverse sections of the segment were made using a freezing microtome (leica cryostat, CM 3000). DAPI (4', 6- Diamidino- 2-phenylindole dihydrochloride) from Vector Laboratories, Inc. (Burlingame, CA) was used for counterstaining to help in the identification of the spinal cells. Finally, the labeled motoneurons were counted with using fluorescent microscopy (Olympus Ax70) in all groups.

### Histological study

Eight weeks after surgery 5 animals from each group were deeply anesthetized and perfused as described above. The embedded spinal cord (L4- L6) was dissected out and left for 2 hours in 2.5 % glutaraldehyde. It was then dehydrated and washed in 0.1 cacodylate buffer and postfixed in 1% osmium tetroxide containing 0.8% potassium ferrocianide and 5 nM calcium chloride in 0.1 M cacodylate buffer for 90min. After washing, samples were stained with 1% uranyl acetate overnight, dehydrated in graded acetone, infiltrated with Poly/Bed 812 resin (Polysciences, Inc., Washington, PA) and polymerized for (60 hours). Ultra thin sections (50-70 nm) were made using an ultramicrotome (Leica ultracut UCT) and then collected on copper grids for transmission electron microscopy (Ziess, EM 900). Intracytoplasmic vacuoles, increased nuclear condensation and marginal chromatin were detected in different groups. 

### Statistical analysis

All Serial 50 µm - thick transverse sections of the L4-L6 segment of the spinal cord from 10 rats were considered in each group. One-way ANOVA and Least Significant Difference (LSD) was used for data analysis. All data are expressed as mean ± SD. A p- value < 0.01 was considered to be statistically significant.

## Results

### Tracing study

**Fig 1:**
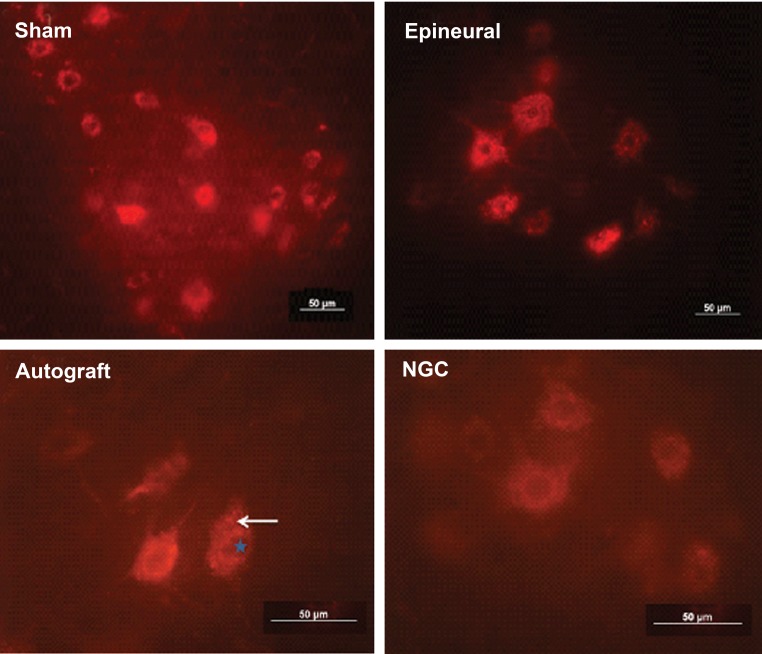
Labeled motoneurons in **L4-L6** segment of the spinal cord in the different groups after eight weeks of treatment. Motor neuron cells in the ventral horn contain red colored particles (arrow) which indicate retrograde DiI vesicles. The nucleus of the nerve cells is seen as a cavity (star). Control and epineural; ×200, autograft and NGC; ×400. Bars: 50 µm .

 In the tracing study, all serial sections of the L4-L6 segment of the spinal cord in each group were examined for retrograde tracing labeled motoneurons. Depending on the level of axonal regeneration, DiI particles were observed to have transferred from the gastrocnemius muscles to the motoneurons in the ventral horn of the lumbar spinal cord(Fig 1).

**Fig 2:**
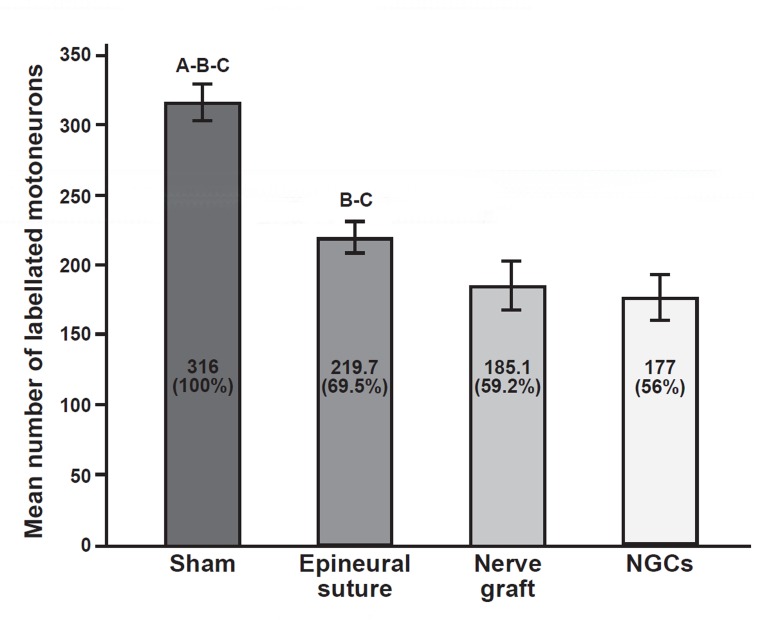
The mean number and mean percent of labeled cells in different groups. There was a significant difference (p<0.01) in the mean number of label cells in the epineural suture group compared to the autograft and nerve guidance channel groups and in the control group compared to the other groups. The number of labelled cells in control rats was considered to be 100%. A. The difference with epineural group is significant (p<0.01), B. The difference with autograft group is significant (p<0.01), C. The difference with NGC group is significant (p<0.01).

 The average number of labeled motoneurons in the control, epineural suture, autograft and nerve guidance channel groups were 316 ± 13.18, 219.7 ± 11.88, 185.1 ± 18.47 and 177 ± 17.13, respectively (Fig 2). The mean number of labeled motoneurons in control animals that had not received transection of the sciatic nerve was considered to be 100% and the other groups were compared to this group. One-way ANOVA and LSD tests showed that there was a significant difference in the mean number of labeled motoneurons in the control animals compared to the other groups (p<0.01).Epineural suture rats, which received transection without removal of a 10 mm section of sciatic nerve, demonstrated more axonal regeneration and transfer of DiI particles from the gastrocnemius muscle to the spinal cells. The mean number of labeled motoneurons in the epineural suture group was significantly different to the autograft and guidance channel groups (p<0.01). More importantly, compared with the control group (100%), axonal regeneration in NGC group (56%) was nearly the same as in the autograft group (59%), suggesting it is a viable substitute for nerve regeneration by autologus graft in the case of a 10 mm sciatic nerve defect.

**Fig 3:**
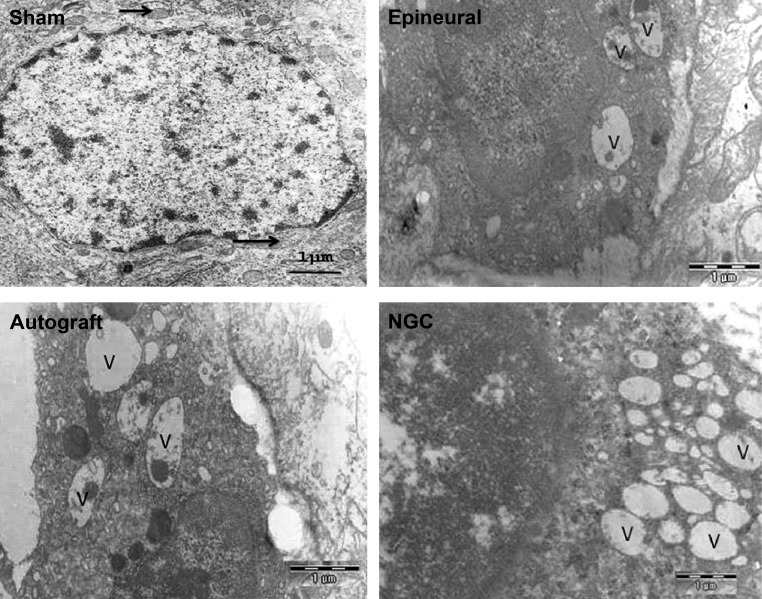
Electron micrograph of spinal motoneuron cells eight weeks after sciatic nerve transection in different groups. In the control group the cells can be seen to have normal mitochondria (arrow) and euchromatin within the cell nucleus (magnification × 12400). A few vacuoles are seen in a nerve cell from the epineural group (magnification × 12000). The apoptotic morphology of the motoneurons with large vacuoles (V) can be observed in the autograft group (magnification ×12000). The number of these vacuoles increased in the NGC group (magnification ×20400). Bars=1 µm.

### Morphological changes

Spinal motoneurons in the control group showed nuclear membrane integration and euchromatin that expanded throughout the nucleus(Fig 3 control).Morphological changes such as, peripheral chromatin condensation was found in epineural suture repair group (Fig 3 epineural).More ultrastructural changes, including dilations of the granular endoplasmic reticulum with large vacuoles were observed in autograft animals(Fig 3 autograft).In addition, the volume of the nucleus reduced in many cells in the autograft group compared to the other groups. In the NGC group the size of the nucleus has seen to be normal but the number of vacuoles, albeit of smaller size, increased(Fig 3 NGC).

Furthermore, central and peripheral chromatolysis was observed in the autograft and NGC groups. Electron microscope observation confirmed the tracing study and demonstrated that the morphological changes in NGC and autograft were closely similar. The ultrastrutural changes were more numerous in the nuclei and cytoplasmic organelles of the autograft and NGC groups compared to the epineural treatment group.

## Discussion

Results from this study have clearly demonstrated the existence of the DiI tracer in the spinal motoneurons of NGC rats, indicating that some axons at the injury site regenerated in the distal portion of the PVDF channel. In addition, these regenerated nerves reinnervated the gastrocnemus muscle and transported retrogradly DiI particles to the spinal cells. Probably the collagen gel and the nerve growth factor within the tube assisted forward regeneration of the fibers. The significant reduction of the labeled motorneurons in the NGC and autograft groups compared to the epineural suture treatment group was due to the 10mm gap of the sciatic nerve. Following nerve transection the retrograde flow of trophic molecules interrupted from the skeletal muscles (target organs) to the cell body of motoneurons in anterior horn of gray matters of the spinal cord can cause morphological changes and cell death in some of them (16-18). In our previous study, charged PVDF with NGF and collagen gel as nerve guidance channel reduced cell death rate at the level of autograft(17) It has been documented that sympathetic and sensory neurons go through cell death after axotomy (14)Probably survival of the nerve cells in the central nervous system is dependent on growth factor from the target cells; brain-derived neurotrophic factor has been shown to rescue spinal motor neurons from axotomy-induced cell death(19). Our ultrastructural findings confirmed the tracing study that charged PVDF with NGF and collagen gel could help the nerve repair and prevent the neural cell death changes. This experimental study demonstrated that PVDF tube in conjunction with efficient therapies could be an alternative to autograft in peripheral nerve defects. It has been shown that the laminin-soaked collagen in a polyglycolic acid (PGA)-collagen nerve conduit supported the nerve repair and functional recovery after grafted into an 80 mm gap in a nerve injury in a dog(20).Although, suturing the epineurium with fascicular opposition after nerve injury can improve the outcome, it will be possible only when the nerve gap is short and the epineurium suture is not accompanied by nerve strain (21). A further consideration is that the sciatic nerve has a weak epineural or perineural structure and the fascicle is changed after transection(21, 22)So, for nerve defects in which a long gap is present, another approach such as nerve guidance or autologus vein graft is needed(23, 8). Our result is consistent with other results showing that the nerve autogrfat could provide a suitable environment for axon regeneration(3). However, on occasions in autulogous nerve graft the nerve is not sufficient in length and diameter for the defect site(24). The advantage of the synthetic tube is that it could be easily manipulated to provide an environment that would encourage regeneration and preserve neuronal morphology. Currently there is an attempting to make a special conduit nerve that could be used together with different therapies to replace the autologus nerve graft and thus eliminate the removal of tissue from the patients. The results of our tracing experiment demonstrated that in NGC animals the PVDF channel with nerve growth factor and collagen gel created a protective sheath for accelerating nerve regeneration. Neurotrophic factor from fibrin matrices enhances sciatic nerve regeneration and insulates the nerve repair site in a long defect by reducing the infiltration of scar tissue(25). As an alternative to nerve autografts these materials have been shown to provide an appropriate environment for the growth of axons from the lesion site and the promotion of direct axonal sprouting by providing a conduit for diffusion of regeneration promoting factors and protecting the regenerating axon from interference by scar tissue(26).

The conduit nerve containing schwann cells with neurotrophic factors and cell adhesion molecules has been shown to promote nerve repair and functional recovery following transection of the sciatic nerve in rats(27).Also, other biological materials such as trophic factors, fibronectin and laminin could be integrated with nerve guidance channels for nerve repair(27-29). 

 PVDF could be used with neurotrophic factors or transplant cells as a delivery device. Promising studies have shown that the incorporation of collagen gels and laminin within guidance channel improve axonal regeneration and functional recovery compared to saline -filled tubes. It has also been reported that peripheral nerve regeneration has been seen following seeded shwann cells in semipermeable guidance channel(29, 30).

## Conclusion

We concluded that, in 10 mm gap of sciatic nerve injury the PVDF tube containing NGF and Collagen gel is a bridge that provide a suitable medium for nerve regeneration and cell death reduction. Further work on peripheral nerve injury with using PVDF channel with other materials is needed to improve our understanding of the best methods for nerve repair.
